# Supplementary material for: Sustained freshening of Arabian Sea High Salinity Water induced by extreme precipitation events
Source: Nat Commun. 2025 Apr 17;16:3667. doi: 10.1038/s41467-025-58950-9 (PMC12006309; doi:10.1038/s41467-025-58950-9)
Supplement: Supplementary file 1 — Supplementary Information [file 41467_2025_58950_MOESM1_ESM.pdf]

Sustained Freshening of Arabian Sea High Salinity Water Induced by Extreme Precipitation Events

Prasad G. Thoppil[1*]

[1]Ocean Sciences Division, U.S. Naval Research Laboratory, Stennis Space Center, Mississippi 39529, USA, *email: prasad.g.thoppil.civ@us.navy.mil

Supplementary Figures

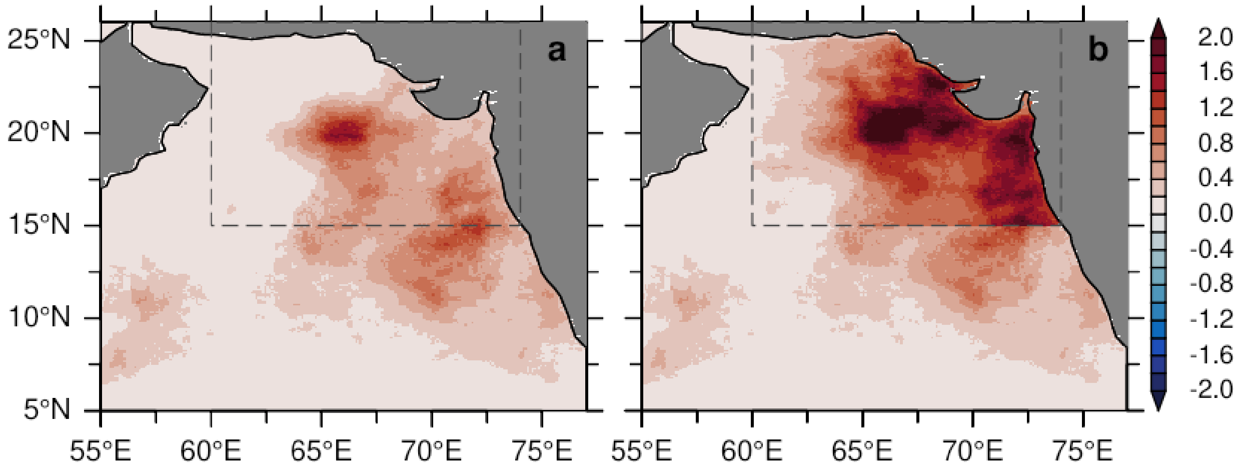

Supplementary Figure 1. Precipitation differences (mm d^{-1}) during 2019–2023: (a) excluding tropical cyclones and (b) excluding both tropical cyclones and extreme precipitation events from 2020 and 2022. These maps illustrate the average freshwater input associated with cyclones and the combined influence of cyclones and extreme precipitation events during 2019–2023. The methodology for isolating precipitation contributions is detailed in the Methods section. Monthly mean precipitation data are sourced from the IMERG dataset. The study region, 60°–75°E and 15°–25°N, is highlighted. These precipitation sources played a key role in the observed salinity freshening during 2019–2023.

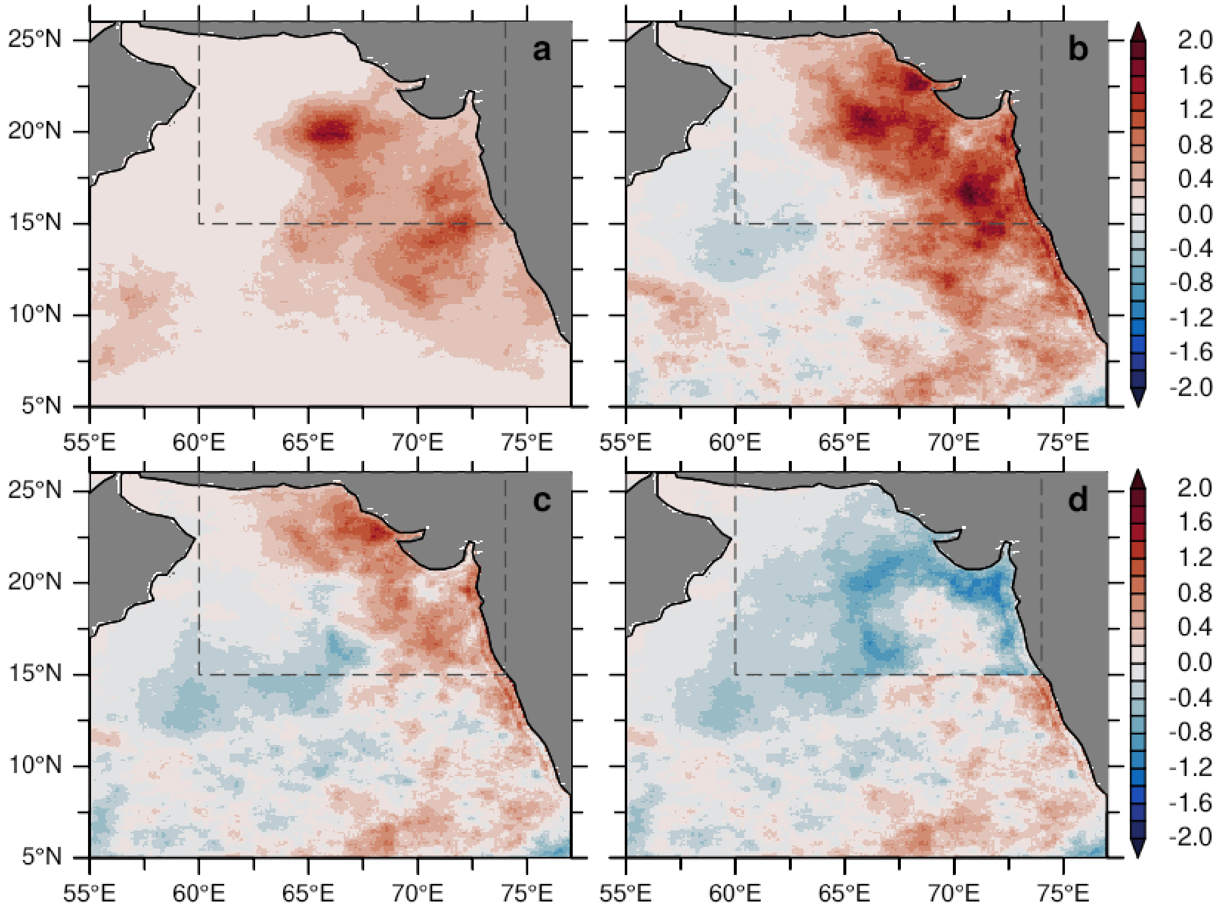

Supplementary Figure 2. Precipitation differences (mm d^{-1}) highlighting the contributions of tropical cyclones and extreme precipitation events during 2019–2023: (a) Precipitation anomalies directly associated with tropical cyclones during 2019–2023. (b) Difference between the 2019–2023 mean precipitation and the long-term average (LTA, 2001–2023). (c) Difference between the 2019–2023 mean precipitation excluding tropical cyclones and the LTA, isolating non-cyclone contributions. (d) Difference between the 2019–2023 mean precipitation excluding both tropical cyclones and extreme precipitation events of 2020 and 2022 and the LTA, highlighting background precipitation patterns (see Methods for separation of precipitation contributions from tropical cyclones and extreme events).

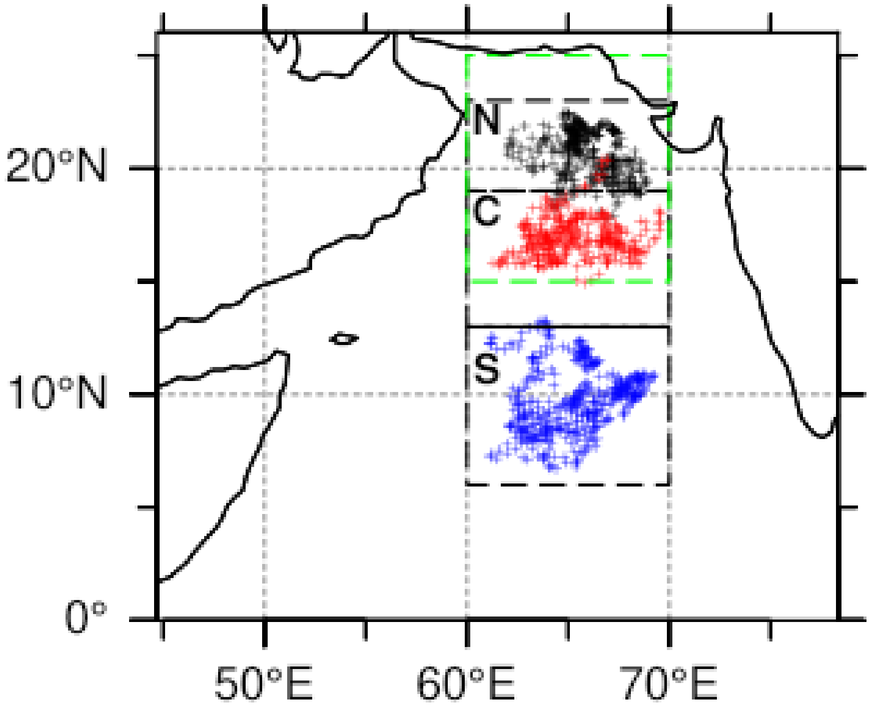

Supplementary Figure 3. Argo float location in the Arabian Sea. Argo float locations in the Arabian Sea during the 2014–2023 period. The Argo observations are grouped into three subregions: North (N, black), Central (C, red), and South (S, blue). The North subregion includes profiles between 19° and 23°N, Central subregion accounts for profiles found between 15° and 19°N, and South subregion accounts for profiles between 6° and 13°N. While a single Argo float may not provide complete coverage for the study period spanning from 2014 to 2023, profiles from additional Argo floats within the same grid boxes were stitched together. Stitching was performed based on observation time, resulting in slight discontinuities due to variations in float locations. Despite this, the stitched data provided seemingly continuous coverage for the entire 2014–2023 period. Along-track salinity displayed in **Figure 3** are from these three N, C, and S regions respectively. The salt-budget terms in **Figure 8** are averaged over the region 60°–70°E, 15°–25°N, as outlined by the green box.

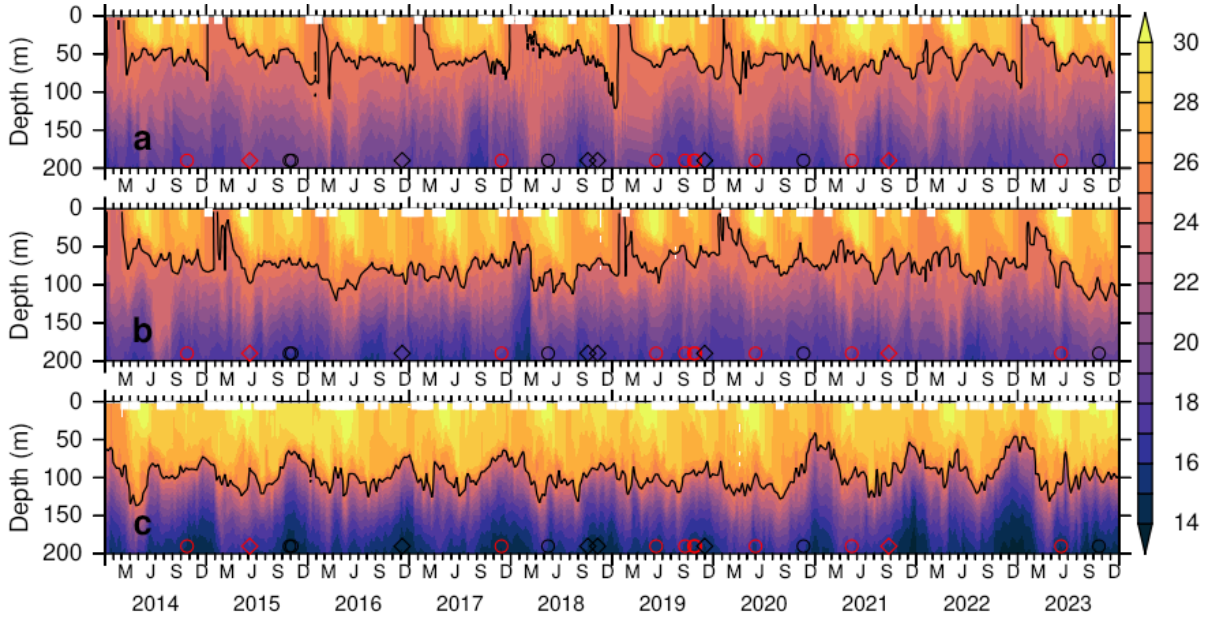

Supplementary Figure 4. Argo temperature observations in the Arabian Sea. Along-track Argo float temperature (°C) observations for three regions representing (a) northern, (b) central, and (c) southern Arabian Sea (refer to Supplementary Figure 3 for location) from 2014 to 2023. The 25°C contour is overlaid in all panels. Profiles with the presence of a barrier layer is marked with white boxes. Barrier layer thickness (BLT, m) is calculated as the difference between the isothermal layer depth (ILD) and the mixed layer depth (MLD), where MLD is defined as the depth corresponding to a density change associated with a 0.2°C temperature difference from the surface, and ILD defines as the depth where the temperature decreases by 0.2°C from the surface. Profiles with BLT ≥ 5 m are marked with white boxes. Cyclone occurrences are marked in each panel: circles indicate very severe to super cyclones, and diamonds indicate cyclones to severe cyclones. Cyclones penetrating north of 15°N are marked in red.

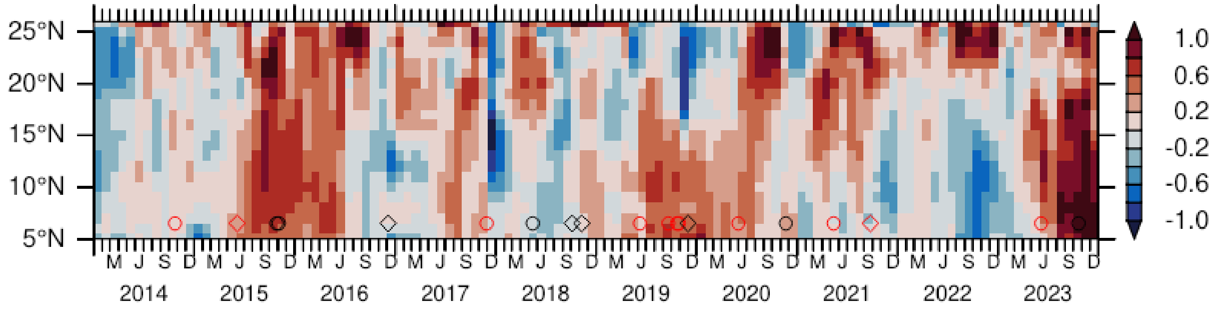

Supplementary Figure 5. Temperature anomalies (°C) averaged in the upper 50 m from the monthly EN4 dataset. Anomalies are calculated relative to the 2000–2023 period by subtracting the long-term monthly climatology. Cyclone occurrences are marked: circles indicate very severe to super cyclones, and diamonds indicate cyclones to severe cyclones. Cyclones penetrating north of 15°N are marked in red.

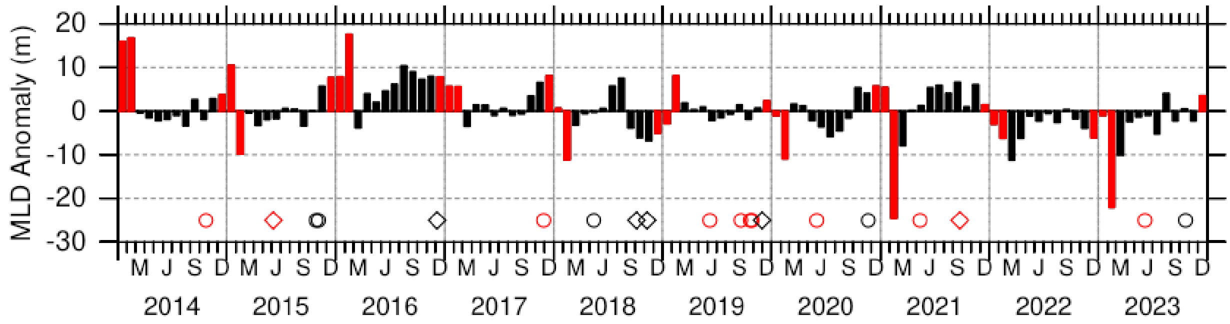

Supplementary Figure 6. Weakening of winter convective mixing. Mixed layer depth (MLD, m) anomaly calculated from the monthly EN4 data averaged for the region 60°–70°E, 15°–25°N. MLD is calculated from the density increase equivalent to a 0.5°C temperature decrease from the surface. Anomalies are calculated relative to the 2000–2023 period by subtracting the long-term monthly climatology. The MLD anomaly during December–February period is highlighted in red. Negative MLD anomalies during winter indicate shallower depth of convective mixing. Cyclone occurrences are marked: circles indicate very severe to super cyclones, and diamonds indicate cyclones to severe cyclones. Cyclones penetrating north of 15°N are marked in red.

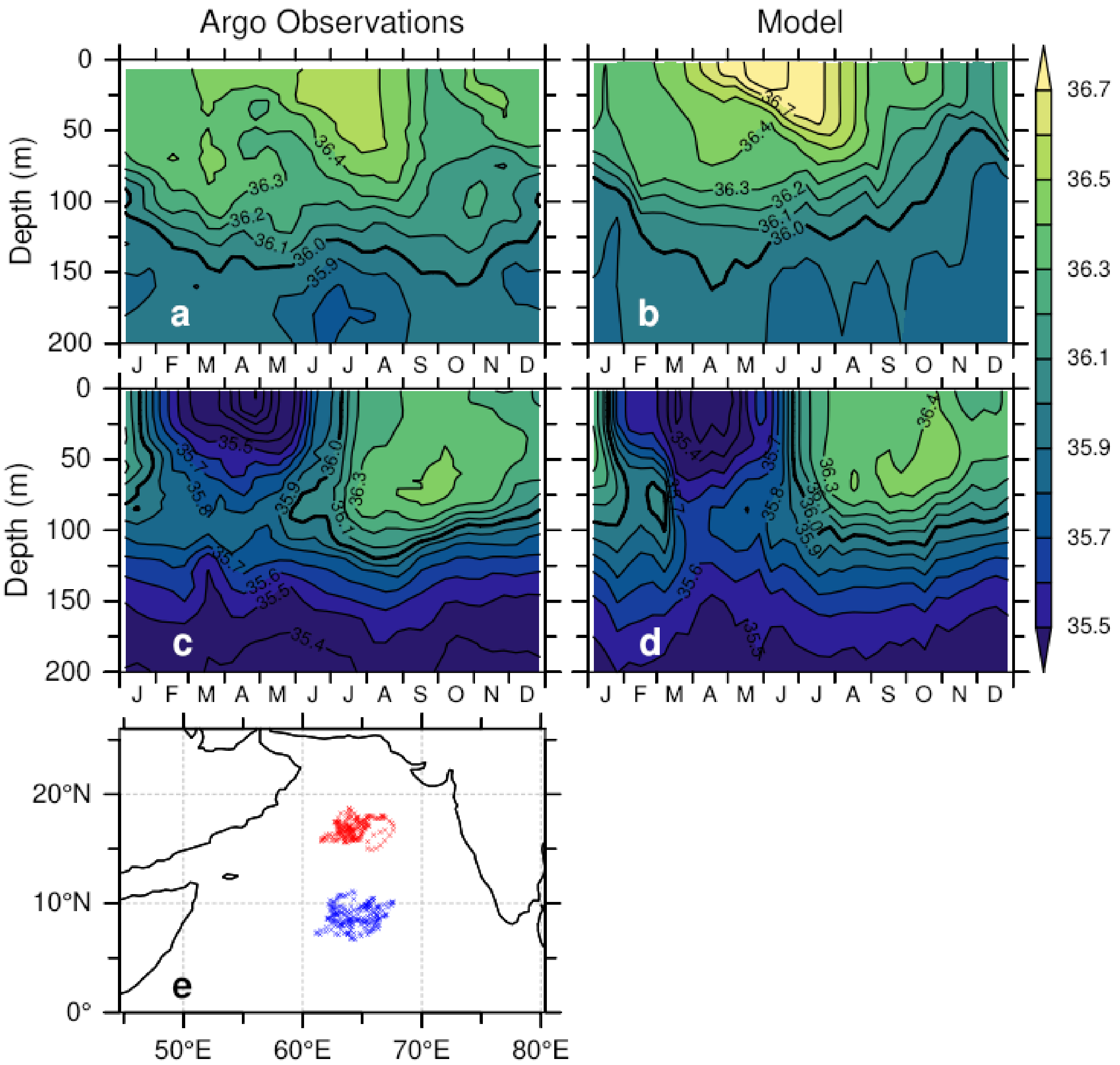

Supplementary Figure 7. Model validation against Argo observations. Climatological salinity (psu) annual cycles are shown for (a, c) Argo observations (b, d) model output, based on two Argo observations: one in the northern (red, *ID = 2902263*) and the other in the southern Arabian Sea (blue, *ID = 2902388*). The along-track salinity is extracted from the model output using a linear interpolation at the (e) Argo geographical locations as indicated by red and blue symbols. With Argo observations occurring on average every 10 days, a 10-day climatology was constructed using six years of data (2018–2023). The locations of the Argo floats (e) in the northern (red) and southern Arabian Sea (blue) are also shown.

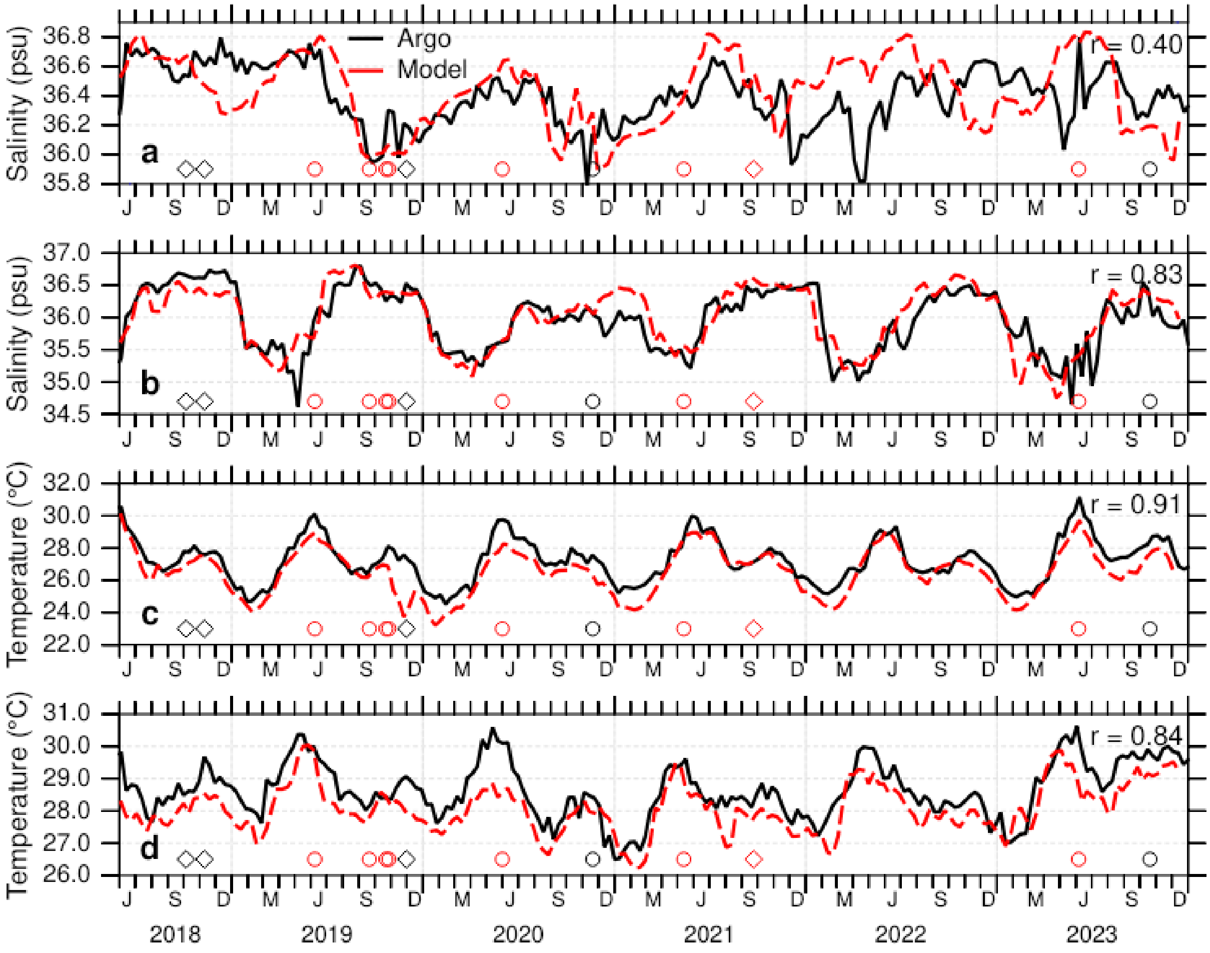

Supplementary Figure 8. Model validation against Argo temperature and salinity observations. Along-track salinity (psu) and temperature (°C) averaged in the upper 50 m for Argo observations (black) model output (red), based on two Argo observations in the (a, c) northern (*ID = 2902263*) and (b, d) southern Arabian Sea (*ID = 2902388*). The along-track salinity and temperature are extracted from the model output using a linear interpolation at the Argo geographical locations shown in **Supplementary Figure 7e**. Cyclone occurrences are marked in each panel: circles indicate very severe to super cyclones, and diamonds indicate cyclones to severe cyclones. Cyclones penetrating north of 15°N are marked in red. The correlation coefficient between the model and observations are shown in each panel.
